# The small heat shock proteins from *Acidithiobacillus ferrooxidans: *gene expression, phylogenetic analysis, and structural modeling

**DOI:** 10.1186/1471-2180-11-259

**Published:** 2011-12-07

**Authors:** Daniela A Ribeiro, Luiz EV Del Bem, Renato Vicentini, Lúcio FC Ferraz, Mario T Murakami, Laura MM Ottoboni

**Affiliations:** 1 Center for Molecular Biology and Genetic Engineering (CBMEG), State University of Campinas - UNICAMP, Candido Rondon Avenue 400, 13083-875 - Campinas, SP, Brazil; 2 National Biosciences Laboratory (LNBio), National Laboratory of Energy and Materials Research (CNPEM), Giuseppe Máximo Scolfaro Street 10000, 13083-970 - Campinas, SP, Brazil

## Abstract

**Background:**

*Acidithiobacillus ferrooxidans *is an acidophilic, chemolithoautotrophic bacterium that has been successfully used in metal bioleaching. In this study, an analysis of the *A. ferrooxidans *ATCC 23270 genome revealed the presence of three sHSP genes, Afe_1009, Afe_1437 and Afe_2172, that encode proteins from the HSP20 family, a class of intracellular multimers that is especially important in extremophile microorganisms.

**Results:**

The expression of the sHSP genes was investigated in *A. ferrooxidans *cells submitted to a heat shock at 40°C for 15, 30 and 60 minutes. After 60 minutes, the gene on locus Afe_1437 was about 20-fold more highly expressed than the gene on locus Afe_2172. Bioinformatic and phylogenetic analyses showed that the sHSPs from *A. ferrooxidans *are possible non-paralogous proteins, and are regulated by the σ^32 ^factor, a common transcription factor of heat shock proteins. Structural studies using homology molecular modeling indicated that the proteins encoded by Afe_1009 and Afe_1437 have a conserved α-crystallin domain and share similar structural features with the sHSP from *Methanococcus jannaschii*, suggesting that their biological assembly involves 24 molecules and resembles a hollow spherical shell.

**Conclusion:**

We conclude that the sHSPs encoded by the Afe_1437 and Afe_1009 genes are more likely to act as molecular chaperones in the *A. ferrooxidans *heat shock response. In addition, the three sHSPs from *A. ferrooxidans *are not recent paralogs, and the Afe_1437 and Afe_1009 genes could be inherited horizontally by *A. ferrooxidans*.

## Background

*Acidithiobacillus ferrooxidans *is an acidophilic, chemolithoautotrophic bacterium that derives energy from the oxidation of ferrous iron, elemental sulfur and reduced sulfur compounds [1]. This bacterium has been successfully used in bioleaching to recover metals from low-grade sulfide ores. During the bioleaching process, *A. ferrooxidans *is subjected to extreme growth conditions, such as temperature increase, pH fluctuations, nutrient starvation, and the presence of heavy metals [2], all of which can affect the efficiency of metal recovery.

Temperature change is one of the most common environmental stresses that can influence essential bacterial processes such as energy transduction and growth. All organisms tend to respond to environmental stresses with a rapid transient increase in heat shock protein (HSP) synthesis. HSPs act either as molecular chaperones, mediating the correct folding and assembly of proteins, or as proteases, irreversibly degrading unfolded proteins [3]. The HSPs are usually classified according to their molecular weights, and the small HSPs (denoted sHSPs) include the categories HSP100, HSP90, HSP70, HSP60, and HSP20.

The sHSPs are characterized by a molecular mass of between 12 and 43 kDa and the presence of 80 to 100 residues that constitute the α-crystallin domain, which is flanked by C- and N-terminals that present lower similarity. The N-terminus is critical to α-HSP activity *in vivo*, playing a role in α-HSP oligomerization and substrate binding [4,5]. The α-crystallin domain is known to possess a molecular chaperone role [6], and the C-terminal extension maintains α-HSP solubility, stability, and chaperone activity [4].

The sHSPs have been extensively studied due to their importance in protecting cellular proteins and maintaining cellular viability under intensive stress conditions, which is particularly important for extremophile microorganisms. Interestingly, most extremophiles posses one or two sHSPs, and species harboring at least 3 sHSP genes are mostly from the Archea domain. However, three sHSP genes have been identified in the genome of *A. ferrooxidans *ATCC 23270 [7].

Xiao et al. [8] showed that there could be significant differences in the expression levels of *A. ferrooxidans *ATCC 23270 sHSP genes in response to heat shock. These findings suggest that *A. ferrooxidans *sHSP genes may be controlled by different regulatory mechanisms, which could be related to specialized functions of the genes. In this study, the expression levels of three sHSP genes (Afe_1009, Afe_1437, and Afe_2172) were investigated in the *A. ferrooxidans *LR strain subjected to heat shock. Phylogenetic analysis and comparative molecular modeling were used to provide new insights concerning the structure and function of the sHSPs from *A. ferrooxidans*.

## Methods

### Bacterial strain and growth conditions

The Brazilian strain *A. ferrooxidans *LR [9] was grown at 30°C and 250 rpm in modified T&K liquid medium [10] containing 0.4 g/L K_2_HPO_4_.3H_2_O, 0.4 g/L MgSO_4_.7H_2_O, 0.4 g/L (NH_4_)_2_SO_4_, and 33.4 g/L FeSO_4_.7H_2_O. The pH was adjusted to 1.8 with sulfuric acid. For the heat shock experiments, *A. ferrooxidans *LR cells were grown in T&K liquid medium until 50% oxidation of Fe^2+ ^was reached. The cells were then collected, inoculated into 100 ml of T&K liquid medium, and incubated at 40°C and 250 rpm for 15, 30 and 60 minutes.

### RNA isolation

The total RNA was isolated from three independent *A. ferrooxidans *cultures, according to the procedure described by Paulino et al. [11]. The cells were suspended in a solution containing 1 mM EDTA, 100 mM LiCl, and 100 mM Tris-HCl, at pH 7.5. The RNA fraction was extracted with phenol/chloroform/isoamyl alcohol (25:24:1, v/v/v) containing 10% (w/v) SDS, precipitated at -20°C with 2% (w/v) potassium acetate at pH 5.5 and 100% (v/v) ethanol, and resuspended in DEPC-treated water. The RNA was treated with DNase (Invitrogen) for 1 h at 37°C, and stored at -70°C.

### Quantitative real-time PCR (qRT-PCR)

The relative expressions of Afe_1009, Afe_1437, and Afe_2172 were determined by qRT-PCR [12]. The cDNAs were synthesized with the ThermoScript RT-PCR system kit (Invitrogen). The *alaS *gene was used as the endogenous control [13]. The primers used in the experiments were designed with the Primer3 program http://frodo.wi.mit.edu/, employing the entire coding region of the selected genes from the *A. ferrooxidans *ATCC 23270 genome (Table 1). The specificity of the primers was confirmed by PCR using genomic DNA from *A. ferrooxidans *LR.

**Table 1:** Primers used in the real-time PCR experiments.

Target gene	Forward primer (5' → 3')	Reverse primer (5 '→ 3')	Amplicon length (bp)
**Afe_1009**	CCGAAATACCTGAGGTCAA	TCCCTTTCTCCTCCTTCTCC	91
**Afe_1437**	GTATTGAAGGCGGAGATTGC	TCTTCTTCCTTGACGCCACT	118
**Afe_2172**	AGGTAATCTTCAGCGGCAAC	TAGGGGATCTCCAGACGATG	97

The qRT-PCR experiments were performed in triplicate using a 7500 Real Time PCR System (Applied Biosystems), and threshold cycle (Ct) numbers were determined using Real Time System RQ Study Software v. 1.3.1 (Applied Biosystems). The qRT-PCR reactions were performed in triplicate using Platinum SYBR Green qPCR SuperMix-UDG (Invitrogen). After thermal cycling, a dissociation (melting) curve analysis was performed to ensure the specificity of the amplifications and the absence of primer-dimer and unspecific amplifications. The relative gene expression was calculated according to the comparative critical threshold method (ΔΔTC) described by Livak and Schmittgen [14]. The statistical significance of the qRT-PCR data was determined using the Student's t-test (p-value ≤ 0.05).

### Bioinformatics analysis

The *A. ferrooxidans *ATCC 23270 genome (J. Craig Venter Institute - http://cmr.jcvi.org/cgi-bin/CMR/Genome) was used to search for genes encoding sHSPs. CLUSTAL W was employed to align the sHSP sequences from *A. ferrooxidans *with sequences found in other bacteria. The alignment was edited with the GeneDoc program [15].

Prediction of the transcription start site was performed with BPROM software (Softberry, Inc.). A widely accepted theoretical informational approach was adopted to identify potential σ^32 ^sites [16,17]. Since the σ^32 ^binding site comprises two conserved blocks (-35 and -10), separated by a gap of variable length, two positional weight matrices (PWM) were generated, each one based on complementary information from the -35 and -10 binding sites. The frequency matrix was based on a set of eighteen *V. cholerae *σ^32 ^promoters [18], including the extended σ^32 ^promoter, with 6 positions in the -35 element and 8 positions in the -10 element, separated by a spacer of variable length. Using the PWMs as a scoring function, putative -35 and -10 regions of σ^32 ^were searched on 200 bases upstream from the ATG start codon of the *A. ferrooxidans *sHSP genes. Each site was scored for its degree of matching to the σ^32 ^-35 and -10 PWMs.

### Phylogenetic analysis

A search was performed against all complete bacterial genomes (1295 genomes on 08/03/2010), using NCBI's microbial genome BLAST tool http://www.ncbi.nlm.nih.gov/sutils/genom_table.cgi?organism=microb and the protein sequences from Afe_1009, Afe_1437 and Afe_2172 as queries. The 20 best hits for each *A. ferrooxidans *sHSP were selected to build an alignment using MAFFT v6.717b http://align.bmr.kyushu-u.ac.jp/mafft/software/. The alignment containing 76 aligned residues was used to produce a maximum likelihood (ML) tree using PhyML 3.0 software http://atgc.lirmm.fr/phyml/. The PAM matrix procedure [19] was used to calculate genetic distances, and statistical support for the nodes employed aLRT statistics [20].

### Molecular modeling

PSI-BLAST search against the Protein Data Bank (PDB) using the three *A. ferrooxidans *sHSPs (Afe_1009, Afe_1437, and Afe_2172) resulted only in templates with low sequence identity (< 28%). However, fold assignment searches using the pGenTHREADER algorithm implemented in the PSIPRED server [21] returned two structures that had significant scores, both of which displayed well-conserved α-crystallin domains. The crystal structures of HSP16.9 from wheat (wHSP16.9, PDB entry code: 1GME) [22] and HSP16.5 from *Methanococcus jannaschii *(MjHSP16.5, PDB entry code: 1SHS) were used as three-dimensional templates for molecular modeling of the α-crystallin domain. The N-terminal region was modeled using only the wHSP16.9 structure as template. Template and target sequences were aligned using the mGenThreader server [23], and were carefully examined to confirm the alignment accuracy. Comparative protein modeling by satisfaction of spatial restraints was carried out using the program MODELLER 9v7 [24]. Fifty models were built for each sHSP from *A. ferrooxidans*, and all models were evaluated with the DOPE potential. Models of each protein with the lower global score were selected for explicit solvent molecular dynamics (MD) simulation, using GROMACS [25] to check for stability and consistency. The overall and local quality of the final model was assessed by VERIFY3D [26], PROSA [27] and VADAR [28]. Three-dimensional structures were displayed, analyzed, and compared using the programs COOT [29] and PyMoL [30].

## Results and Discussion

### The sHSPs from *A. ferrooxidans*

Search of the *A. ferrooxidans *ATCC 23270 genome (J. Craig Venter Institute) revealed the presence of three sHSP genes (Afe_1009, Afe_1437, and Afe_2172) belonging to the HSP20 family. According to Han and co-workers [31], about 71% of the microbial organisms with completed annotated genomes possess one or two sHSP genes, and 10% of the Archaea species have more than three sHSP-related genes. Notably, the genome of *Bradyrhizobium japonicum *(a rhizobial species) possesses 13 sHSP-related genes [32].

Laksanalamai and Robb [7] showed that the degree of identity of the sHSPs from several extremophiles possessing only one sHSP was 75%, while the identity of sHSPs from the same organism ranged from 20 to 50%. The low sequence identity for the *A. ferrooxidans *sHSPs (Table 2) is therefore intriguing.

**Table 2:** Physical and chemical parameters of the three sHSPs from *A. ferrooxidans*.

Gene	Length	Molecular weight (Da)	Theoretical pI	Identity/similarity to Afe_1009	Identity/similarity to Afe_1437	Identity/similarity to Afe_2172
**Afe_1009**	145	16934	6.20	-	29/58%	26/47%
**Afe_1437**	148	16680	5.43	29/58%	-	22/53%
**Afe_2172**	134	16401	5.60	26/47%	22/53%	-

Afe_1009, Afe_1437, and Afe_2172 are not organized in an operon in the *A. ferrooxidans *genome. Indeed, most of the known sHSP genes are not arranged in operons [33,34], with some exceptions such as the *Escherichia coli ibpAB *operon, which contains two sHSP genes (*ibpA *and *ibpB*) [35,36], and *Bradyrhizobium japonicum*, which has sHSP genes found as independent units and others grouped in the same operon [32].

### sHSP genes expression in *A. ferrooxidans *LR cells subjected to heat shock

**Figure 1:**
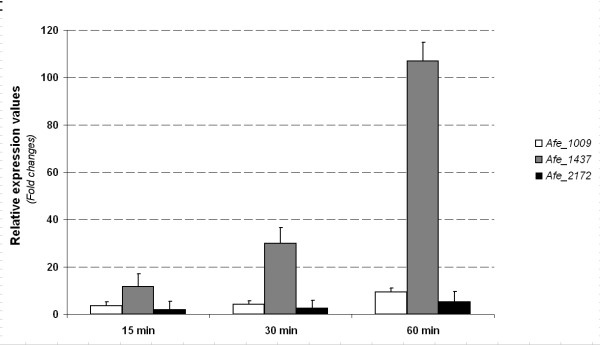
**Expression of the *shsp *genes from *A. ferrooxidans *LR**. Expression of the genes located at loci Afe_1009, Afe_1437, and Afe_2172 in *A. ferrooxidans *LR cells submitted to heat shock (40°C) at different times (15, 30, and 60 min). The expression values, obtained by Real time PCR, are relative to the ones obtained from cells maintained at 30°C.

qRT-PCR was used to determine the transcript levels of the Afe_1009, Afe_1437, and Afe_2172 genes in *A. ferrooxidans *LR cells grown at 30°C (control) or subjected to a 40°C heat shock for 15, 30 and 60 minutes (Figure 1). The qRT-PCR results indicate that after 60 minutes all three sHSP genes were significantly up-regulated (p < 0.05 and fold change ≥ 2.0), although the expression level of Afe_2172 was considerably lower than the expression levels of Afe_1437 and Afe_1009. The expression level for Afe_1437 was 20-fold higher than that observed for Afe_2172, and 11.5-fold higher than the expression level of Afe_1009. Xiao et al. [8] observed a similar pattern of expression for the Afe_1437 gene. Our results for Afe_1009 and Afe_2172 were dissimilar to those obtained by Xiao et al. [8]. However, this comparison may not be reliable due to differences in the *A. ferrooxidans *strains as well as the heat shock experiments used in the two studies.

**Figure 2:**
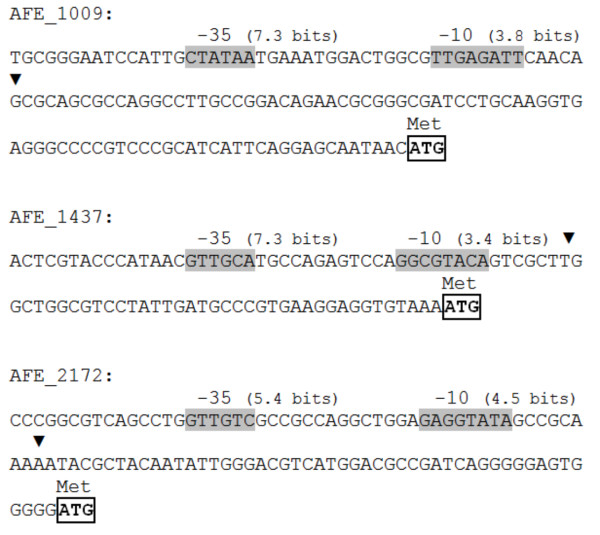
**Nucleotide sequences of the 5'-upstream regions of the three genes, Afe_1009, Afe_1437, and Afe_2172, which encode sHSPs in *A. ferrooxidans***. Transcription start sites predicted by the BPROM program and promoter sequences recognized by the σ^32 ^factor are indicated by black triangles and by shadowed-bold letters, respectively. The first codon of the coding sequence is indicated by boxed letters. The total information content of the σ^32 ^boxes (-35 and -10) is shown in bits.

The observed differences in the expressions of the three *A. ferrooxidans *sHSP genes suggest possible regulatory differences. In many bacteria, the σ^32 ^factor regulates the expression of the sHSP-encoding genes in a temperature-dependent manner [35]. Under stress conditions, the transcription of heat shock genes is induced following a rapid and transient increase of this factor [37]. A bioinformatics analysis was therefore performed in the deduced -10 and -35 regions of the three sHSP genes. The results indicated that the three genes had possible σ^32^-dependent promoters (Figure 2). In the work undertaken by Xiao et al. [8], σ^32^-dependent promoters were only found for the Afe_1437 and Afe_2172 genes. However, the disparities between the two studies can be explained by the different *in silico *strategies chosen.

In *A. ferrooxidans*, the -35 motif at the σ^32 ^binding site appears to be more conserved than the -10 motif. The same occurs for the *V. cholerae *and the *E. coli *σ^32 ^consensus sequences [18]. In spite of the different expression levels observed for the *A. ferrooxidans *sHSP genes, the bioinformatics analyses did not reveal any other type of regulation mechanism (data not shown). However, within the σ^32^-regulated genes, alternative mechanisms of regulation are possible. Münchbach and co-workers [32] used subtractive two-dimensional gel electrophoresis to identify a set of 10 sHSPs in *B. japonicum *subjected to a temperature shift from 28°C to 43°C. These authors observed that the amounts of the sHSPs were quite dissimilar, suggesting the existence of a diverse regulatory repertoire.

### Phylogenetic analysis and comparative sequence analysis

**Figure 3:**
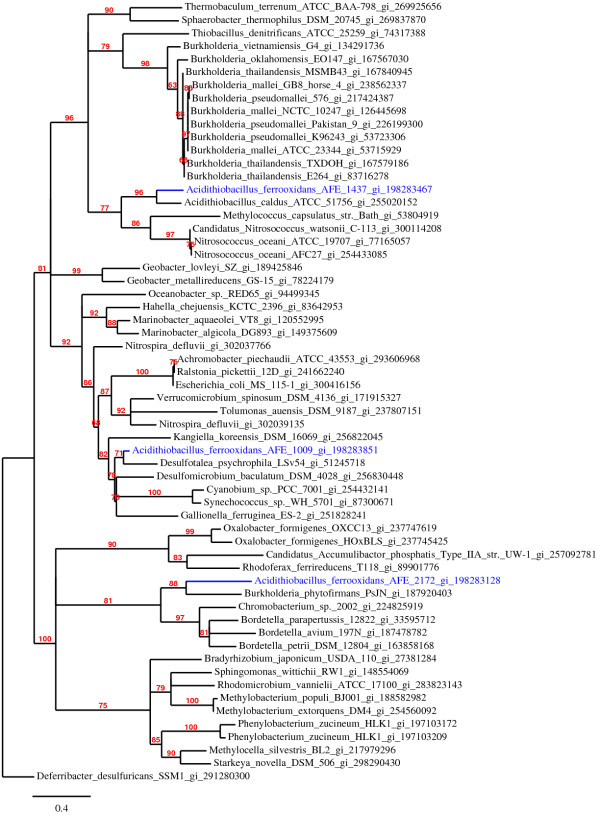
**Inferred phylogenetic relationships among the *A. ferrooxidans *and closely related bacterial sHSPs**. The 20 closest related bacterial protein sequences to each *A. ferrooxidans *sHSP were retrieved by a BLAST search against 1295 completed bacteria genomes (see Methods section). The topology was obtained by ML using 76 aligned amino acids residues. Distances were calculated by PAM matrix and the statistical confidence of the nodes was calculated by aLRT test. Branches with aLRT values lower than 50% were collapsed. GeneBank accession numbers are shown in front of the species name.

The ML analysis suggested that the three sHSPs from *A. ferrooxidans *are not recent paralogs (Figure 3). This finding is in accordance with the low sequence similarity between the sHSPs from *A. ferrooxidans *(Table 2 and Figure 3). The sequence divergence among the *A. ferrooxidans *sHSPs is likely to be the consequence of horizontal transfer of one or even two genes; however, the possibility of divergent evolution [38] caused by different selective pressures cannot be fully discarded. To gain more insight into the origins of the *A. ferrooxidans *sHSPs, the CG content of each gene was compared with the average CG content of *A. ferrooxidans *coding-genes (~59% of CG). The CG contents of Afe_1437 (46.53%) and Afe_1009 (47.71%) were statistically different from the average *A. ferrooxidans *CG content (p < 0.01; x^2 ^= 11.7766 and x^2 ^= 9.4510, respectively), while for Afe_2172 (58.76%) there was no significant difference (x^2 ^= 0.1025). These findings suggest that Afe_1437 and Afe_1009 could be inherited horizontally by *A. ferrooxidans*. Interestingly, the closely related species *A. caldus *from the same genus has only one sHSP gene, which is the possible ortholog of *A. ferrooxidans *Afe_1437. Considering the hypothesis of horizontal transfer origins of Afe_1437 and Afe_1009, it is likely that *A. caldus *has lost the ortholog of Afe_2172 (putative original sHSP) and maintained the ortholog of Afe_1437. In this scenario, the lateral transference that originated Afe_1437 occurred prior to the divergence between these two species.

**Figure 4:**
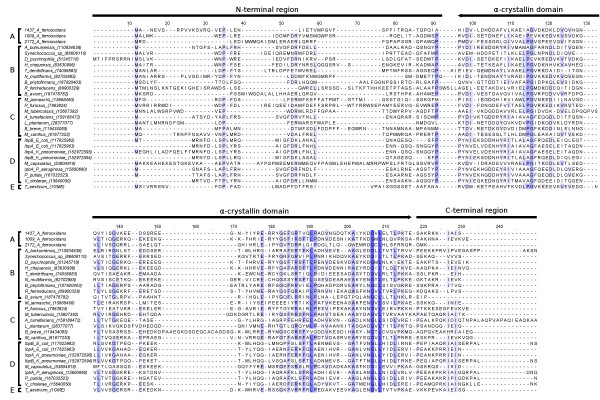
**Alignment of the protein sequences of the sHSPs from *A. ferrooxidans *and other bacteria**. Sequences were grouped as follows: Group A, the amino acid sequences from the *A. ferrooxidans *sHSPs; Group B, sHSP sequences from phylogenetically related species; Group C, sHSPs with three-dimensional structure established and with chaperone activity characterized; Group D, sHSPs with chaperone activity from gamma-proteobacteria; Group E, the amino acid sequence from the well-characterized sHSP from *Triticum aestivum*.

Figure 4 shows the alignment of the amino acid sequences of the three sHSPs from *A. ferrooxidans *with other sHSP sequences, including sequences from the gamma-proteobacteria subdivision. As shown in Figure 4, the sHSPs from *A. ferrooxidans *harbor the well-conserved α-crystallin domain and all elements considered essential for their oligomerization, and therefore for their chaperone activity. However, the Afe_2172 protein has a very short C-terminus that is rarely observed in sHSPs from other bacteria. The only other exception is a sHSP from *Bordetella avium*, a bacterium that causes an upper respiratory tract disease in avian species (Figure 4). This feature can either decrease their ability to oligomerize or modulate their chaperone activity. Moreover, the C-terminal region of sHSPs from some bacteria presents highly conserved cysteine residues. These residues have been proposed to enable the sHSPs to sense changes under oxidizing conditions of the environment, and to translate these changes into differences in protein conformation and chaperone activity [39]. Also, in some plant species, a conserved methionine-rich sequence at the N-terminal region has been proposed to offer a redox control of chaperone-like activity and dynamics of the oligomeric structure [40]. However, these conserved cysteine residues at the C-terminus, as well as the conserved methionine-rich motif at the N-terminus, were not found in the sHSPs phylogenetically related to *A. ferrooxidans *(Figure 4), which suggests an absence of such control in the sHSPs belonging to the gamma-proteobacteria subdivision.

The N-terminal region showed no significant sequence similarity to other sHSPs with well-defined chaperone activity (groups C and D), but secondary structure prediction tools indicated that all of the sequences analyzed had the propensity to form the α-helical structures that are considered key elements for substrate binding and stabilization of the oligomeric structure. Furthermore, the N-terminal region alone was capable of interacting with denatured proteins [41], and its truncation reduces the chaperone activity of sHSPs [42]. These findings emphasize that this region contains the substrate binding site, and is therefore important for the chaperone activity.

### Structural modeling of the sHSPs from *A. ferrooxidans*

*In silico *three-dimensional models of the proteins encoded by Afe_1009, Afe_1437, and Afe_2172 displayed excellent global and local stereochemical properties, with a Z-score (PROSA server) of around -3.5 and all residues lying within the allowed regions of the Ramachandran plot. A good Z-score means that it is within the range of scores typically found for native proteins of similar size. RMSD analysis of the template crystal structures and the developed models resulted in values below 0.5 Å for the main-chain backbone of the α-crystallin domain, suggesting that the models were suitable for structural and comparative analyses.

**Figure 5:**
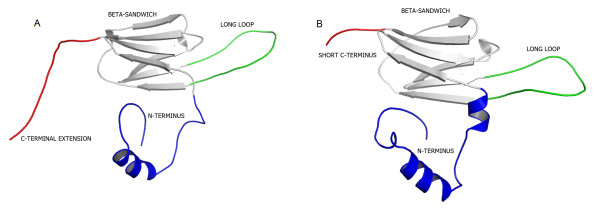
**Cartoon representation of the modeled structure of the sHSPs from *A. ferrooxidans***. (A) Proteins encoded by loci Afe_1009 and Afe_1437. (B) Protein encoded by loci Afe_2172. The b-sandwich domain, long loop, and N- and C-terminal regions are colored in light grey, green, dark blue, and red, respectively.

The α-crystallin domains of the proteins encoded by Afe_1009, Afe_1437, and Afe_2172 share similar structural features with other sHSPs from both prokaryotic and eukaryotic organisms. This domain (residues 46-135) shows a β-sandwich fold composed of seven β-strands in two sheets (Figure 5). The N-terminal region (residues 1-45), encompassing two helical segments, was only observed in the structure of wHSP16.9 from wheat [22]. In the wHSP16.9 structure, the N-terminal helices participate in the stabilization of the oligomeric structure, establishing interactions with the adjacent α-crystallin domain [22]. The C-terminal extension (136-148) displays a random coil conformation and has a critical role in the formation of the oligomeric state. However, different to the proteins encoded by Afe_1437 and Afe_1009, the Afe_2172 protein has a rare shortened C-terminus, which may prevent the formation of a stable oligomer and could be involved in the modulation of the protein chaperone activity. Canonically, the long loop, which is responsible for dimerization, is fully conserved, and the identification of functional regions by surface-mapping of phylogenetic information, using the ConSurf web server [43], indicates that all residues considered essential for dimerization are fully conserved in the three sHSPs from *A. ferrooxidans*.

In order to gain insights into the oligomeric state of the proteins encoded by Afe_1437 and Afe_1009, which possess the extended C-terminus, analysis was performed of the structural determinants required for assembling into either a dodecameric double disk (wHSP16.9) or a spherical shell composed of 24 monomers (MjHSP16.5). In both the wHSP16.9 and the MjHSP16.5 structures, the intermolecular interactions made by the C-terminal extension are virtually identical, despite the fact that the C-terminus of wHSP16.9 requires two different orientations to form the oligomer. This ability of the C-terminus to adopt two conformations resides in the amino acid segment between the strands β 9 and β 10, which permits a hinge movement. Analysis of the C-terminus contacts in the MjHSP16.5 structure showed that the segment between the strands β 9 and β 10 adopts a conformation stabilized by hydrogen bonds between the OεGlu137 and NεGln52 atoms, and the carbonyl oxygen of the Glu137 and NζLys142 atoms. Surprisingly, these contacts are not found in the wHSP16.9 structure, due to the presence of a second Pro residue at position 142 that enables the segment to fold into a stable motif, generating a 6-residue segment (KAEVKK) with high flexibility, which allows the hinge movement. In both Afe_1437 and Afe_1009 protein sequences, this segment does not contain a proline residue at the same relative position, and the residues populating this segment have all the requirements to form a stable motif in the same way as the MjHSP16.5 structure. Thus, based on our structural findings, we suggest that both Afe_1437 and Afe_1009 proteins behave like the prokaryotic sHSP from *M. jannaschii*, adopting a 24-molecule hollow spherical shell. However, additional experimental data obtained using techniques that can provide insights into hydrodynamic behavior, such as dynamic light scattering, ultra-centrifugation, size-exclusion chromatography and small angle X-ray scattering, are required to confirm our *in silico *predictions.

## Conclusions

In this study, we have demonstrated that the expression level of the *A. ferrooxidans *Afe_1437 gene is considerable higher than that of the Afe_2172 gene, and that the three sHSP genes harbor possible σ^32^-dependent promoters. The three sHSPs from *A. ferrooxidans *are not recent paralogs, while the genes Afe_1437 and Afe_1009 can be inherited horizontally by *A. ferrooxidans*. This suggests that the sHSPs encoded by Afe_1437 and Afe_1009 are more likely to act as molecular chaperones in the *A. ferrooxidans *heat shock response. These findings were corroborated by molecular modeling showing that both Afe_1437 and Afe_1009 proteins behave like the prokaryotic sHSP from *M. jannaschii*, a well characterized sHSP with chaperone activity.

## Authors' contributions

All authors have read and approved the final manuscript. DAR and LMMO conceived the idea and designed the experiments. DAR and LFCF executed the RTq-PCR experiments. DAR wrote the manuscript. RV performed the bioinformatics analysis; LEVDB, the phylogenetic analysis; and MTM, the molecular modeling.
